# Transposon-derived genome editors in plants: from compact nucleases to large-fragment integration and regeneration strategies

**DOI:** 10.48130/els-0026-0001

**Published:** 2026-06-15

**Authors:** Xuemin Jiao, Wenhua Chen, Zongyou Lv, Wansheng Chen

**Affiliations:** 1 Research and Development Center of Chinese Medicine Resources and Biotechnology, Shanghai University of Traditional Chinese Medicine, Shanghai 201203, China; 2 Department of Pharmacy, Changzheng Hospital, Second Military Medical University, Shanghai 200003, China; 3 State Key Laboratory for Quality Ensurance and Sustainable Use of Dao‐di Herbs, Beijing 100700, China; 4 State Key Laboratory of Discovery and Utilization of Functional Components in Traditional Chinese, Institute of Chinese Materia Medica, Shanghai University of Traditional Chinese Medicine, Shanghai 201203, China

**Keywords:** Transposon-derived editors, TnpB, IscB, Fanzor, Plant regeneration

## Abstract

CRISPR/Cas technology has been transformative for genome editing, enabling the precise integration of large DNA fragments—a capability essential for advanced genome (re)writing in plants, such as for trait stacking and pathway engineering. However, several new RNA-guided systems derived from transposable elements (TEs) have shown promise for plant genome editing, thereby enriching the toolkit available for plant engineering. This review comprehensively analyzes these emerging TE-based editors, including OMEGA nucleases (TnpB, IscB, Fanzor), CRISPR-associated transposases (CASTs), and R2 retrotransposon–derived systems. We detail their distinct architectures, evolutionary origins, and unique advantages over traditional Cas9, such as their compact size, simplified guide RNAs, and staggered DNA cleavage. We critically evaluate their nascent applications in plant models, highlighting ongoing challenges in editing efficiency, delivery, and specificity. Notably, CASTs and R2 retrotransposon–derived systems show particular promise for programmable large-fragment DNA integration, offering potential solutions for next-generation plant genome writing applications. We also examine the critical link between genome editing and plant regeneration, discussing phytohormonal and morphogenic regulators (e.g., BBM, WUS), and innovative tissue-culture-free methods, such as Cut-Dip-Budding. By comparing their merits and limitations, we position these systems not as replacements but as complementary tools within an expanding genome-editing toolkit. Integrating optimized TE-based editors with robust regeneration strategies is poised to unlock new frontiers in plant synthetic biology and crop improvement.

## Introduction

The advent of clustered regularly interspaced short palindromic repeats (CRISPR)/CRISPR-associated nuclease 9 (Cas9)-based genome editing has democratized targeted genome manipulation, revolutionizing the life sciences^[1,2]^. In plants, it has enabled efficient gene knockouts, base editing, and modulation of transcriptional activity, accelerating both functional genomics and the development of new crop traits^[3]^. Despite these advances, a central challenge remains largely unresolved: the precise, efficient, and scarless integration of large DNA fragments (> 1 kb), such as full-length genes, multigene cassettes, or synthetic metabolic pathways^[4]^. This capability, often termed 'genome writing', is essential for advanced applications such as trait stacking, *de novo* metabolic engineering, and the creation of domesticated-like varieties from wild accessions in a single generation.

Transposable elements (TEs) are among the potential tools for genome writing. These ubiquitous genomic parasites encode the molecular machinery required to excise and reinsert themselves into host genomes, sometimes with passenger DNA alongside. For decades, TEs like *Sleeping Beauty*^[5]^, derived from fish genomes, and *PiggyBac*^[6]^, derived from the moth cabbage looper (*Trichoplusia ni*), have been used as semi-random integration tools to engineer the genomes of animal cells. TEs in plants play important roles in modulating gene expression and driving genome evolution and crop domestication^[7]^. In bacteria, transposons mainly function as an RNA-guided defense system against invading viruses. Insertion sequence 200 (IS200) and IS605 superfamily transposons, like the CRISPR/Cas9 system, incorporate protospacers into CRISPR-like arrays and generate spacers when their host is infected by a virus^[8]^. The spacers are then transcribed from the CRISPR arrays and loaded by the CRISPR RNA (crRNA) and transactivating CRISPR RNA (tracrRNA), thereby targeting the viral genome for degradation by Cas effectors^[8−10]^. Because the mechanism behind the recognition of foreign sequences by the IS200/IS605 transposons is similar to that of the CRISPR/Cas9 system, they can be developed as gene-editing tools for animals^[11]^and plants^[12]^ by changing the obligate mobile element-guided activity (OMEGA) RNA (ωRNA). Consequently, transposon-derived programmable nucleases, including TnpB^[8,9]^, IscB^[8]^, and Fanzor systems^[10]^, mPing^[13]^, CRISPR-associated transposases (CASTs)^[14,15]^, and R2 retrotransposon–derived systems^[16]^, have significantly enriched the toolkit available for plant engineering.

As different editing tools have different merits and each tool can functionally complement the limitations of others, transposon-based systems can expand the breadth of genome editing. Here, we provide an overview of the use of transposon-based systems to develop gene-editing tools for plant functional genomics and genome engineering^[17,18]^, and discuss some of their applications in diverse plant models^[19,20]^. Finally, we discuss the outstanding challenges in this field and propose future directions for development and applications of transposon-based systems in plant and agricultural science.

## Classification and distribution of TEs

Gene-editing applications of transposons were first reported in 2021^[8,9]^. Since then, compact transposon systems for gene editing have generally been divided into three types—type I TnpB, type II IscB, and type III Fanzor—according to their evolutionary relationships^[8]^ (Table 1).

**Table 1:** Summary of compact transposon-encoded editors.

Characteristics	Cas9	TnpB and IscB	Fanzor	Plant genome editing
Size	~1,000–1,400 aa (e.g., SpCas9: 1,368 aa)	~400–500 aa (TnpB); ~350–450 aa (IscB)	~400–600 aa	Delivery advantage: easier packaging into size-limited vectors (e.g., AAV, plant viral vectors); suitable for multiplex editing
Guide RNA	crRNA + tracrRNA (or fused sgRNA)	Single ωRNA (simpler structure)	Single ωRNA (eukaryotic origin)	Simplified design: easier expression and optimization in plants
PAM specificity	Stringent (e.g., SpCas9: NGG)	Relaxed (e.g., TTN, NG)	Moderately relaxed (e.g., T-rich)	Expanded targeting scope: enables editing of genomic regions previously inaccessible due to PAM constraints
Cleavage pattern	Blunt-ended DSB	Staggered DSB (5-nt overhang)	Staggered DSB	Potential for precise integration: staggered ends may facilitate homologous recombination or specific repair pathways
System origin	Bacterial CRISPR immune system	Prokaryotic transposon (IS200/IS605)	Eukaryotic transposon (first eukaryotic-derived programmable nuclease)	Eukaryotic compatibility (Fanzor): may reduce plant cytotoxicity and holds potential for organellar genome editing
Immunogenicity	High (bacterial origin; may trigger plant immune responses)	Potentially lower (still prokaryotic-derived)	Likely lowest (eukaryotic origin; more 'familiar' to plant cells)	Improved editing efficiency: reduced suppression by plant defense responses
crRNA, CRISPR RNA; tracrRNA, transactivating CRISPR RNA; PAM, protospacer adjacent motif; DSB, double-strand break; aa, amino acid.				

### TnpB endonucleases

*TnpB* is carried by IS200/IS605 transposons and constitutes the largest group of genes in prokaryotes, with more than one million putative loci^[8]^. For decades, it was annotated as encoding a putative transposase component, although its precise function remained unclear, as these elements typically mobilize via a distinct transposase (TnpA). In 2021, two groups reported that TnpB can cleave DNA in animal cells^[8,9]^.

Meanwhile, comparative genomic and phylogenetic analyses began to unravel the deep evolutionary connection between certain transposon-encoded proteins, such as TnpB and IscB, and CRISPR/Cas systems^[8]^. In fact, the large Cas9 and Cas12 nucleases have been proposed to have evolved from smaller, transposon-associated ancestors, specifically pointing to proteins like TnpB and IscB as their potential progenitors^[8]^. TnpB-mediated cleavage is guided by a noncoding RNA termed omegaRNA (ωRNA), which could be reprogrammed to cleave specific DNA targets *in vitro* and achieve genome editing in human cells. This work established TnpB as the founding member of a new, minimal class of programmable editors, now classified as OMEGA systems^[8,9]^. Strong phylogenetic and structural evidence indicate that TnpB is the direct evolutionary ancestor of the larger, more complex Cas12 (previously named Cpf1 [CRISPR from *Prevotella* and *Francisella* 1]) family of CRISPR/Cas effectors. Cas12 likely evolved through the fusion of a TnpB-like nuclease with additional protein domains, acquiring new regulatory features and becoming integrated into the adaptive CRISPR immune apparatus. TnpB is closely related to IscB, another IS200/IS605-encoded protein. IscB is even smaller than TnpB and is hypothesized to be the ancestor of Cas9. Thus, TnpB and IscB represent two distinct lineages from which the major CRISPR/Cas nuclease families (Cas12 and Cas9, respectively) originated.

**Figure 1:**
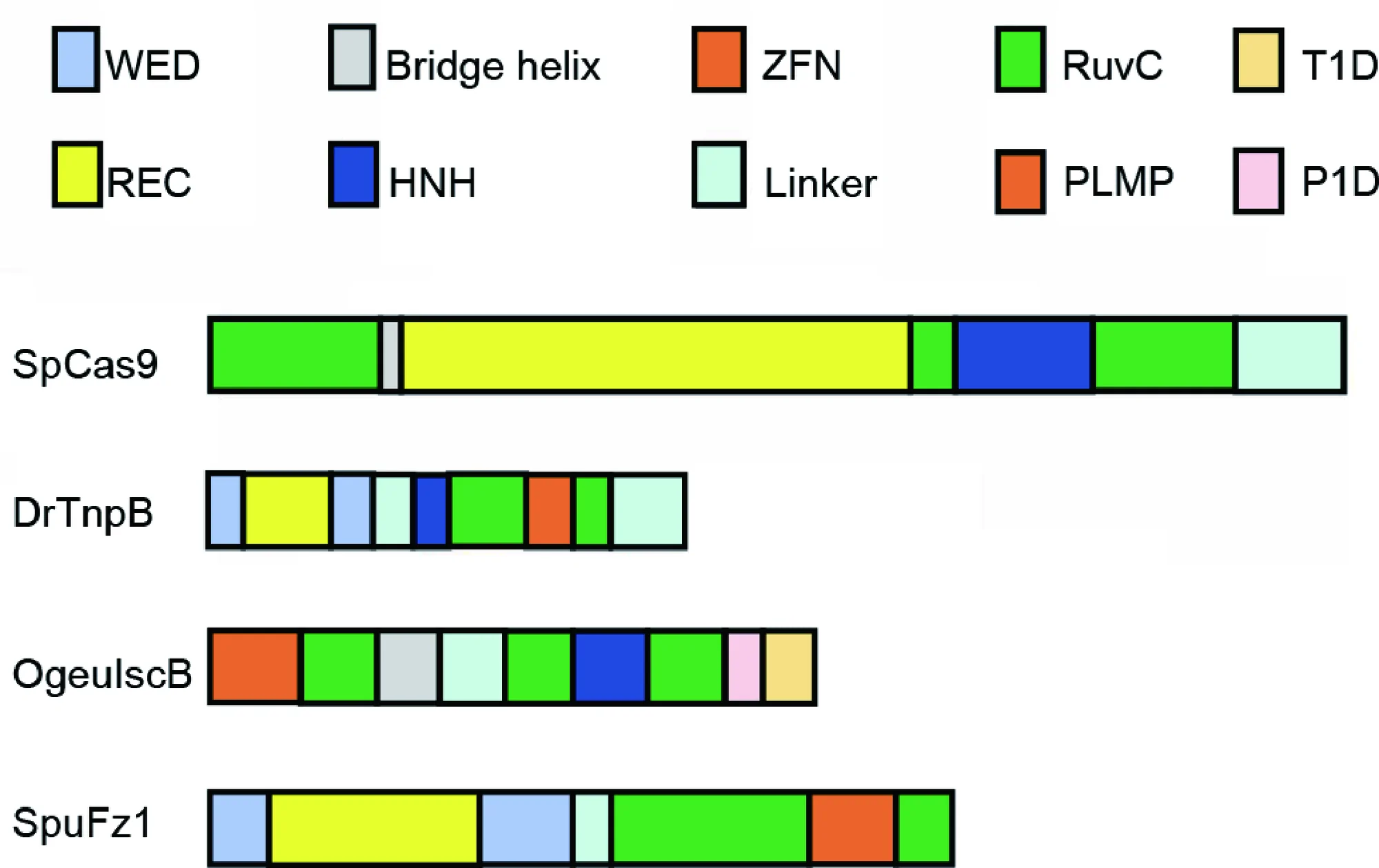
Common structural domains of Cas9, TnpB, IscB, and Fanzor. WED, wedge domain; REC, recognition domain; RuvC, RuvC domain; ZFN, zinc-finger domain. All sequences are drawn approximately to scale.

Genes encoding TnpB homologs are widely distributed across diverse bacterial and archaeal phyla, frequently associated with various mobile genetic elements. This observation suggests that the RNA-guided DNA cleavage activity of TnpB and IscB may have originally evolved to facilitate the mobility or homing of their associated transposons, a function later co-opted for adaptive immunity in CRISPR/Cas systems. Structural studies have revealed that TnpB has two primary lobes, namely the REC (Recognition) lobe and the NUC (Nuclease) lobe (Fig. 1)^[21]^. The REC lobe is responsible for binding to the ωRNA scaffold and facilitating the recognition of the target DNA–ωRNA heteroduplex (Fig. 1)^[21]^, and contains key motifs for interacting with the guide–target hybrid. The NUC lobe contains the catalytic center of the enzyme. It contains a single RuvC-like nuclease domain (Fig. 1)^[21]^, which is structurally and mechanistically related to the RuvC domain found in Cas12 and other RNase H superfamily nucleases. This domain is responsible for cleaving both strands of the target DNA, generating a double-strand break (DSB) with staggered ends (often 5–8 nt 5' overhangs)^[21]^, a signature distinct from the blunt ends produced by Cas9.

TnpB has a low editing efficiency in animal^[9,21]^ and plant cells^[12]^. To improve editing efficiency, many optimizations have been made. For example, a truncated TnpB (ISDra2 from *Deinococcus radiodurans*) with a shortened C-terminal domain (CTD) may achieve genome-editing efficiencies ranging from 45% to 78% in animal cells^[22]^. Further stepwise optimization of the stem 2 region of the noncoding RNA (ωRNA, also called right-end element RNA [reRNA]) component of the ISDra2 system significantly elevated the nuclease activity of ISDra2^[23]^ by enhancing the delivery of ISDra2–ωRNA to the target site and raising the editing efficiency to about 10% in mice (*Mus musculus*)^[23]^. In addition, mutagenesis of the ωRNA scaffold can increase the editing efficiency two-fold. Combined with a deep mutational scan of TnpB, the editing efficiency for insertion/deletions (InDels) reached a high frequency across multiple loci (23%–42%) in human cells, surpassing that of both wild-type ISDra2 and the variant ISYmu1 TnpB (11%–29% and 6%–30%, respectively)^[24]^. Although optimizing TnpB and the ωRNA can clearly enhance editing efficiency many fold, exploring alternative tools is another potential way to increase editing efficiency. For example, a systematic screen of the IS605 transposon family uncovered TnpB orthologs with robust editing activity in human cells. The establishment of a dedicated annotation pipeline enabled the identification of 14 candidate systems, among which ISTfu1, ISDge10, and ISAba30 were shown to efficiently edit dozens of loci when expressed in human cells^[25]^ (Table 2).

**Table 2:** Summary of compact transposon-based systems used in plants.

TnpB	PAM or TAM	Species	Engineering strategy	Mutation efficiency	Spot	Ref.
ISYmu1	TTGAT	*Arabidopsis* protoplasts and plants	Wild type	0.1%–4.2% in protoplasts; 8.5% in plants	Delivery with tobacco rattle virus; transgene-free editing	[30]
ISYmu1	TTGAT	Rice	Wild type	6.3%–4.5%	Produces edited plants with no off-target mutations	[27]
Ymu1	TTGAT	*Arabidopsis* plants	Mutated amino acids	8.2%–24.1%	Virus-mediated delivery without Tissue culture	[46]
eTnpBc	TTGAT	Tobacco	Mutated amino acids	100%	Virus-mediated delivery without Tissue culture	[31]
eTnpBe	TTGAT	pepper	Mutated amino acids	10%	High editing efficiency	[24]
eTnpBe	TTGAT	rice	Mutated amino acids	29.3%	High editing efficiency	[24]
eTnpBc	TTGAT	Tobacco	Mutated amino acids	55%	High editing efficiency	[24]
ISDra2	TTGAT	*Arabidopsis* protoplasts	Wild type	0–4.8%	–	[30]
ISDra2	TTGAT	Rice	Wild type	5.6–41.9%	Produces edited plants with no off-target mutations	[27]
ISDra2	TTGAT	Rice	Wild type	33.6%	Produces edited plants	[26]
ISAam1	TTTAA	*Arabidopsis* protoplasts	Wild type	0%–0.3%	–	[30]
ISAam1	TTTAA	Rice	Wild type	4.5%–6.3%	–	[27]
ISAam1(N3Y)	TTTAA	Soybean hairy roots	Mutated amino acids	1.5%	–	[47]
ISAam1(T296R)	TTTAA	Soybean hairy roots	Mutated amino acids	1.3%	–	[47]
TnpB	TTGAT	*Arabidopsis*	Wild type	14%	Works in multiple species	[12]
IsDge10	TTAT	Rice	Mutated amino acids	4.2%–25%	Multiple sites edited	[28]
enIscB	TGCTAA	Rice	Combination of mutated IscB and ωRNA	41.9%	Fused to T5E	[44]
enIscB	CAGGAA	Rice protoplasts	Mutated amino acids and truncated ωRNA	2.05%–8.3%	–	[28]
SpuFz1	CATA	Rice protoplasts	Wild type	0	No activity observed	[28]
NlovFz2-STU	CCG	Rice	Uses STU	2.8% in rice protoplast; 22.5%–50.0% in rice	Uses the enhanced editing efficiency of STU	[48]
SpuFz1-STU	CATA	Rice	Uses STU	1.4% in rice protoplasts; 24.8%–34.6% in rice	Uses the enhanced editing efficiency of STU	[48]
MmeFz2-STU	TAG	Rice	Uses STU	0.9% in rice protoplasts; 17.8%–18.8% in rice	Uses the enhanced editing efficiency of STU	[48]
GtFz1-STU	TTAAN	Rice protoplasts	Uses STU	0	–	[48]
PAM, protospacer adjacent motif; TAM, target adjacent motif; STU, single-transcript unit.						

Due to the inherent challenges of delivering transgenes into plants, research on transposon editing in plant systems lags behind analogous work in animal systems. Lv and colleagues showed that TnpB can function as a nuclease and edit the genome in the model plant *Arabidopsis* (*Arabidopsis thaliana*), as well as in medicinal plants, such as sweet wormwood (*Artemisia annua*), red sage (*Salvia miltiorrhiza*), Chinese skullcap (*Scutellaria baicalensis*), Chinese woad (*Isatis indigotica*), and Dangshen (*Codonopsis pilosula*)^[12]^. Mutation of TnpB enhanced its editing efficiency in *Nicotiana benthamiana*, pepper (*Capsicum annuum*), and rice (*Oryza sativa*), with an up to a 50-fold higher efficiency than that of wild-type TnpB^[24]^. ISDra2 displayed high editing efficiency in rice, reaching up to 33%–40%^[26,27]^. IsDge10, another TnpB, can generate edits at multiple sites in rice, with mutation efficiency ranging from 4.2% to 25%^[28]^.

The small size of TEs enables their delivery via virus-based vectors. For this reason, once introduced into plant cells, these vectors can produce edited offspring and transgene-free seedlings^[29]^. In support of this, a tobacco rattle virus (TRV)-based vector carrying the compact ISYmul system was shown to generate transgene-free edited plants in *Arabidopsis*^[30]^. Notably, the eTnpBc variant of ISDra2 TnpB further demonstrates the potential of this approach, achieving editing efficiencies of up to 100% in offspring^[31]^.

While the initial editing efficiency of TnpB in eukaryotes was inferior to that of established CRISPR/Cas systems, targeted engineering of its protein stability, ωRNA guide, and expression context has yielded variants with dramatically improved performance. These optimization efforts have transformed TnpB from a biological curiosity into a practical genome-editing tool, valued for its small size and unique biochemical properties. Continued structural and mechanistic studies, coupled with combinatorial engineering approaches, are expected to further refine TnpB, potentially unlocking its full potential for applications where size and specificity are paramount, including multiplexed editing and viral vector–based delivery.

### IscB endonucleases

IscB is also encoded by the IS200/IS605 family of TEs^[32]^. This type of transposon is transposed via a mechanism known as 'peel-and-paste, cut-and-copy'^[33]^. IscB transposases recognize and bind to subterminal palindromic sequences (inverted sequence left end [isLE] and inverted sequence right end [isRE])^[34]^, which are structural motifs characterized by stem-loop configurations located at the termini of transposons. These enzymes catalyze the precise excision of the transposon fragment from the lagging DNA strand, yielding circular single-stranded DNA (cssDNA) intermediates^[9]^. Concurrently, the ends of the flanking donor DNA are rejoined without generating sequence alterations^[9]^. The cssDNA intermediates are subsequently integrated into a new genomic target site, thereby completing the transposition cycle^[9]^.

For years, the function of IscB remained obscure, and it was annotated merely as a hypothetical transposon-associated protein. Advanced comparative genomics later proposed that large Cas nucleases (like Cas9 and Cas12) evolved from smaller, transposon-encoded ancestors, with IscB postulated as the direct progenitor of Cas9^[8]^. This model was validated in 2021 when IscB was shown to function as an RNA-guided DNA endonuclease, dependent not on a CRISPR array but on a dedicated, *cis*-encoded noncoding RNA termed ωRNA^[8,9]^.

IscB proteins are among the smallest known programmable nucleases (typically 350–450 amino acids [aa])^[8,25]^, offering a minimal scaffold for tool development. Structurally, IscBs have a special domain, namely a PLMP-motif containing domain at their N terminus (Fig. 1) that is absent in Cas9^[35]^. However, IscBs lack a polypeptide-based REC lobe^[35]^ and a NUC lobe^[35]^ (Fig. 1). Cleavage typically generates a staggered DSB with a short 5' overhang. The ωRNA itself contains a scaffold region for protein binding and a 5' spacer sequence conferring target specificity.

The editing efficiency of wild-type IscB is suboptimal when expressed in animal cells^[8,9]^, which constrains its utility for *in vivo* applications. However, engineered IscB systems (containing an engineered ωRNA) may approach an editing efficiency comparable to that of Cas9 in animal cells^[36,37]^. The editing efficiency of IscB was significantly enhanced through rational engineering of its protein structure and of its ωRNA guide. Truncation of the R1 stem-loop within the ωRNA by five nucleotides (nt) resulted in an approximately two-fold higher activity^[36]^. Similarly, replacing A–U base pairs with more stable G–C pairs in the ωRNA stem regions improved its structural stability and likewise doubled editing efficiency^[36]^. Most notably, structure-guided mutagenesis of IscB, combined with these optimized ωRNA variants, synergistically elevated editing efficiency to approximately 60%^[23]^. Further optimization of IscB was achieved through structure-guided protein engineering. The introduction of three key substitutions increased its editing efficiency by approximately 7.5-fold^[37]^. Additionally, fusion of a non-sequence-specific DNA-binding domain to IscB further enhanced its editing efficiency, reaching 91.3% in animal cells^[37]^. As with Cas9, optimization of the nuclear localization signal (NLS) represents an effective approach to enhance editing efficiency in plant systems^[38−40]^. In animal cells, nuclear import of IscB was improved by fusing an engineered NLS from simian virus 40 (SV40) to both the N terminus and C terminus of IscB. This modification significantly improved its editing efficiency, achieving an approximately 8.2-fold improvement compared to the unmodified variant^[37]^.

To further modulate editing outcomes, the introduction of deletions can be promoted by fusing exonucleases to endonucleases such as Cas and IscB, which often results in a higher frequency and increased size of deletions. Notable examples include the fusion of nucleases to Three prime repair exonuclease 2 (TREX2), the T5 exonuclease (T5E), the T7 exonuclease (T7E), Exonuclease III (Exo3), and UL12^[41]^. When IscB was fused to T5E, the resulting chimeric protein exhibited an editing efficiency comparable to that of SpCas9 in human cells^[36]^.

In plants, IscB shows a low editing efficiency (Table 2). In rice protoplasts, an engineered IscB (enIscB) achieved editing efficiencies of 2.05%–8.3% at five target sites^[28]^. To improve the editing efficiency, T5E was fused to the C terminus of different mutant variants of enIscB^[37,42,43]^ (enIscBs-T5E); these fusion proteins were combined with different truncated ωRNAs^[37,42,43]^. When these combinations were introduced into rice protoplasts, the editing efficiency rose 5.9–9.7-fold over that of the wild-type IscB–ωRNA system^[44]^, consistent with previous results in mammalian cells^[37,42,43]^.

IscB represents a fascinating and underexplored platform for plant genome editing. While its current editing efficiency in plants is a limiting factor^[28]^, the optimization blueprint established for IscB provides a clear and actionable path forward^[44]^. Through combinatorial engineering of mutated variants^[37,42,43,45]^, addition of an NLS^[38,39]^, fusion to an exonuclease^[11]^, and guide RNA design^[45]^, the activity of IscB can be significantly augmented. As engineering efforts mature, IscB is poised to transition from a biological curiosity into a valuable, niche-specific tool within the arsenal of plant genome-editing tools, complementing existing CRISPR/Cas and transposon-derived systems.

### Fanzor IscB endonucleases

The recent discovery of Fanzor (Fz) proteins, the first known eukaryotic RNA-guided DNA endonucleases, has opened up transformative avenues for genome engineering in plants and animals^[10,49,50]^. First annotated in 2013 as transposon-associated proteins encoded by TEs present in eukaryotic genomes^[51]^, Fzs were functionally characterized in 2023 as programmable nucleases that use ωRNA guides^[10]^.

Phylogenetically, Fzs evolved from bacterial TnpB ancestors^[8,51]^, the same proteins that gave rise to Cas12, through horizontal gene transfer into eukaryotic genomes. This evolutionary history is reflected in their functional domains, which include a conserved RuvC-based nuclease lobe and a variable REC lobe for ωRNA binding^[10]^.

Fz systems are classified within the obligate mobile element-guided activity (OMEGA) superfamily, which includes prokaryotic nucleases like TnpB and IscB^[10]^. Phylogenomic analysis revealed that Fzs form two major clades: Fz1 (widely distributed in fungi, plants, and arthropods) and Fz2 (frequently found in giant viruses and marine eukaryotes)^[10,52]^. Both groups likely originated from independent horizontal transfers of genes encoding TnpB-like proteins into eukaryotic lineages, where they acquired introns, an NLS, and other eukaryotic adaptations.

Fzs are compact nucleases (typically 400–700 aa) consisting of a REC lobe and an NUC lobe. The REC lobe contains the REC and WED domains and binds the ωRNA guide, thereby facilitating target DNA recognition. The NUC lobe consists of the RuvC catalytic domain and the NUC domain responsible for DNA cleavage^[10]^ (Fig. 1). Unlike Cas9, Fz utilizes a single ωRNA, which includes a scaffold region for protein binding and a 5′ spacer sequence for DNA targeting^[10]^. The RuvC domain generates staggered DNA DSBs with 5′ overhangs, similar to TnpB and Cas12^[10]^. This cleavage pattern may favor certain DNA repair pathways in plants, such as microhomology-mediated end joining (MMEJ).

Since the initial report of DNA cleavage by Fz in animal cells in 2023^[10,52]^, with a very low editing efficiency of 0.5%–15%^[52]^, optimized Fz systems have helped enhance editing efficiency^[10]^. Several factors constrained efficiency; one solution was to engineer a better ωRNA: adding a 275-nt extension at the 5' end of the canonical ωRNA enhanced efficiency about 7-fold in human cells^[10]^. Another solution was protein engineering: mutating several amino acids in Fz resulted in a 5-fold higher InDel efficiency compared to the wild-type Fz^[10]^. By combining these mutations with an optimized ωRNA, the editing efficiency of Fz reached 10%–25% for the introduction of InDels at the tested loci^[10]^.

For further optimization, AlphaFold3 was used to predict the structure of Fz2 and engineer a more active nuclease. When used together with an ωRNA stabilized by truncation and mutation of the distal ends of stem loops S1, S2, and S3, these variants exhibit an almost 5-fold increase in genome-editing efficiency compared to wild-type Fz2 combined with unmodified ωRNA^[50]^. In addition, substituting several aa with arginine enhanced the interaction between the RNA-guided nuclease and nucleic acids, thereby improving genome-editing activity in eukaryotic cells^[53,54]^. Five substitutions with arginine in NlovFz2 (from the amoeba *Naegleria lovaniensis*) yielded more than a 20% improvement in editing efficiency compared to wild-type NlovFz2^[50]^. Engineered NlovFz2 substantially improved editing efficiency, achieving an 11.1-fold increase over the wild-type NlovFz2^[50]^.

Limited studies have directly tested the editing potential of Fz in plants (Table 2), but available data suggest that its editing efficiency is lower than that of established CRISPR systems. For example, SpuFz1 from the fungus *Spizellomyces punctatus* showed no detectable editing activity in rice protoplasts^[28]^. Single-transcript unit (STU) technology enhanced editing efficiency in previous studies^[55,56]^. Using STU, the editing efficiency of NlovFz2 in rice protoplasts reached an average efficiency of 2.8%, while SpuFz1 and MmeFz2 (from the mollusk *Mercenaria mercenaria*) achieved an average efficiency of 1.4% and 0.9%, respectively; no activity was detected for GtFz1^[48]^. NlovFz2 and SpuFz1 displayed a similar editing efficiency in wheat (*Triticum aestivum*) protoplasts, with an average efficiency of 2.9% and 1.0%, respectively. In transgenic rice plants, the highest editing efficiencies were achieved by NlovFz2 (22.5%–50.0%), followed by SpuFz1 (24.8%–34.6%) and MmeFz2 (17.8%–18.8%)^[48]^.

Fz-mediated genome editing in plants remains at an early stage of exploration. The established Fz STU system for rice shows promising adaptability for extension to other plant species. With further optimization, Fz-based editors may emerge as a versatile platform for crop trait enhancement, synthetic biology, and functional genomics, offering a complementary approach to existing CRISPR-based technologies.

## Hybrid strategies for targeted transposition

### CRISPR-associated transposases (CASTs)

CRISPR-associated transposases (CASTs) are natural gene-editing systems that combine the programmable DNA-targeting capability of CRISPR/Cas with the efficient DNA integration machinery of transposons. These systems, often referred to as 'cut-and-paste' editors, enable the insertion of large DNA fragments (up to 10 kb) without relying on host DNA repair pathways such as homology-directed repair (HDR). Two kinds of CAST systems have been identified in diverse bacterial lineages. The most well-characterized systems include CAST from *Vibrio cholerae* (VchCAST)^[14,57]^, a Type I-F CRISPR/Cas system associated with a Tn7-like transposon, and CAST from *Scytonema hofmanni* (ShCAST), a Type V-K CRISPR/Cas system utilizing Cas12k also linked to a Tn7-like transposition mechanism^[15,58]^. Both systems represent natural fusions where CRISPR modules guide transposition to specific genomic loci.

CASTs were first systematically characterized in 2019^[14,15]^, when independent research teams discovered that certain Tn7-like transposons had co-opted CRISPR/Cas systems for their targeted integration^[14,15]^. This finding represented a paradigm shift: from the native role of CRISPR systems in adaptive immunity to a mechanism facilitating horizontal gene transfer. Evolutionarily, CASTs appear to have emerged through the domestication of CRISPR/Cas systems by transposons, with the transposon-encoded TniQ protein serving as the molecular bridge that physically links the CRISPR targeting complex to the transposition machinery. This arrangement contrasts with other RNA-guided systems (e.g., TnpB, Fz) that evolved from different transposon lineages.

**Figure 2:**
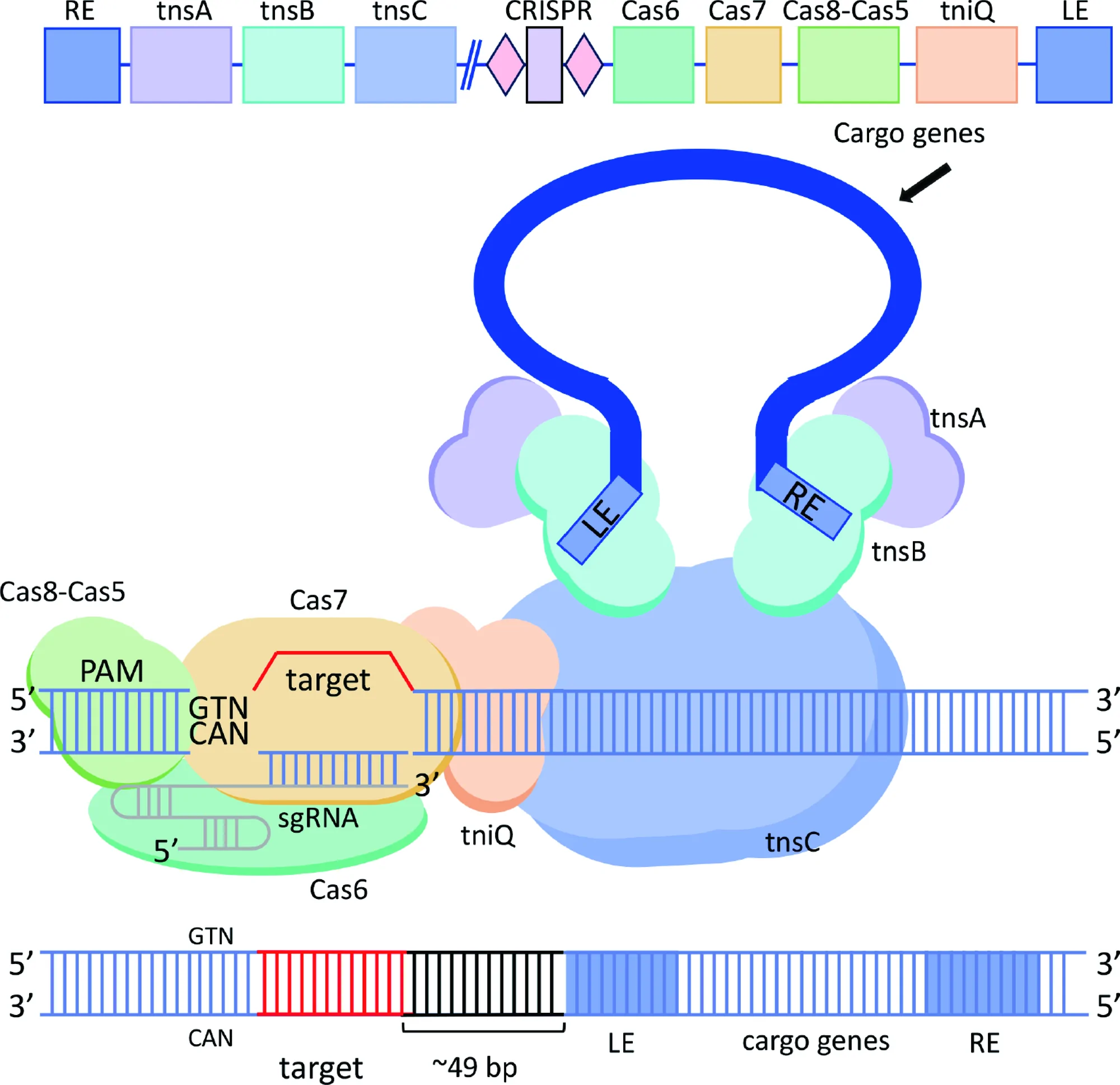
Model of excision and targeted insertion of CRISPR/Cas systems (types I-F). RNA-guided TniQ–Cas complexes recruit the non-sequence-specific DNA-binding protein TnsC to the target site, inducing the excision of the transposon from its donor site and its targeted integration at a fixed distance downstream of DNA-bound TniQ–Cas.

**Figure 3:**
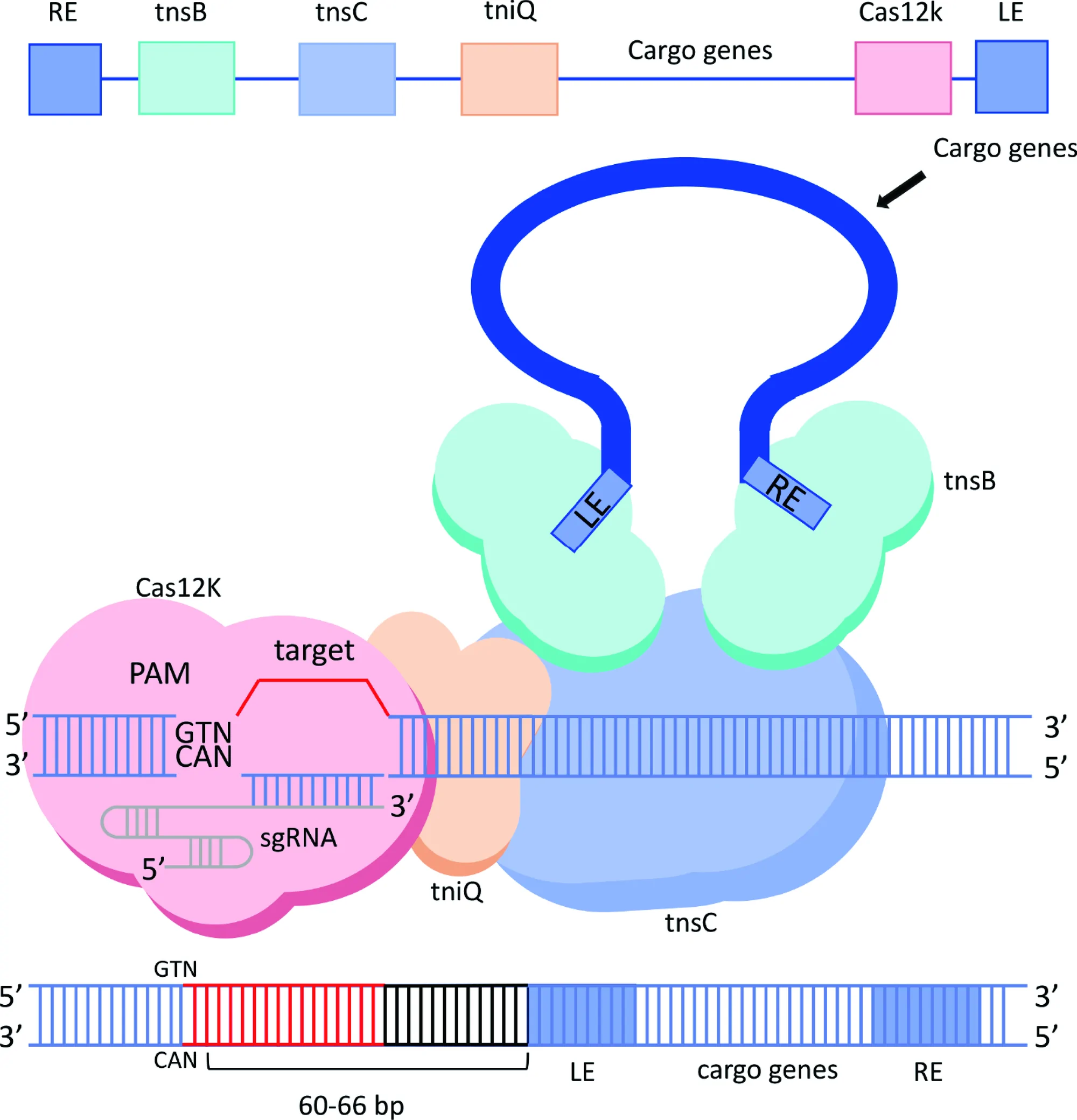
Model of excision and targeted insertion of CRISPR/Cas systems (types V–K) . The Cas12k–TnsB–TnsC–TniQ complex directs the integration of donor DNA 60–66 bp downstream of the protospacer-adjacent motif (PAM). The inserted sequence, flanked by left-end (LE) and right-end (RE) transposon terminal repeats, results in a 5-bp target-site duplication upon integration.

The CRISPR/Cas targeting module is responsible for sequence-specific DNA recognition: in Type I-F systems, this module consists of the Cascade complex (Cas6, Cas7, Cas8, and a crRNA) together with TniQ^[14,57]^ (Fig. 2), whereas in Type V-K systems, it comprises Cas12k, TnsB, TnsC, TniQ, and a crRNA^[15]^. The transposase subunit (TnsA/TnsB) catalyzes the excision of the donor DNA from the vector and generates DSBs^[14]^ (Fig. 3). Specifically, TnsB, a member of the retroviral integrase superfamily, mediates DNA integration, while TnsC acts as an ATPase that coordinates the interaction between TnsAB and the targeting module^[14,57]^. TniQ, a homolog of *E. coli* TnsD, recruits the non-sequence-specific DNA-binding protein TnsC to the target site^[14]^. The donor DNA is flanked by specific transposon ends (*attL* and *attR*) that are recognized by TnsB^[14]^.

In bacterial systems, CAST achieves integration efficiencies exceeding 80% for inserts of several kb in length^[15,58]^. By contrast, reported efficiencies in human cells remain below 5%^[57,59,60]^, highlighting a substantial performance gap that likely reflects challenges in the delivery, expression, and functional coordination of a multi-component prokaryotic system within a eukaryotic environment. A team overcame bottlenecks for CAST integration activity in human cells using phage-assisted continuous evolution (PACE). The evolved system supports ~10%–25% integration efficiencies for 15 kb cargoes in human cells, representing an average ~200-fold improvement in integration activity^[61]^.

CAST systems have not yet been successfully applied in plants; the limitation likely stems from several interconnected challenges: (1) difficulty in balancing the expression of multiple protein components; (2) inefficient simultaneous nuclear import of all subunits; (3) delivery constraints due to large coding capacity; and (4) suboptimal plant regulatory elements and chromatin compatibility. These issues suggest that the current lack of CAST activity in plants is not due to inherent incompatibility, but rather to a lack of system optimization. Future breakthroughs will require advances in multi-component expression, delivery, and protein engineering.

CASTs nevertheless represent a promising solution for precise, large-fragment DNA integration, although their application remains nascent in animal and microbial systems and has not yet been established in plants. While their low efficiency, largely due to delivery and compatibility limitations, currently constrains their use, ongoing advances in protein engineering, expression optimization, and delivery platforms are rapidly addressing these barriers.

### Transposase-assisted target-site integration (TATSI)

**Figure 4:**
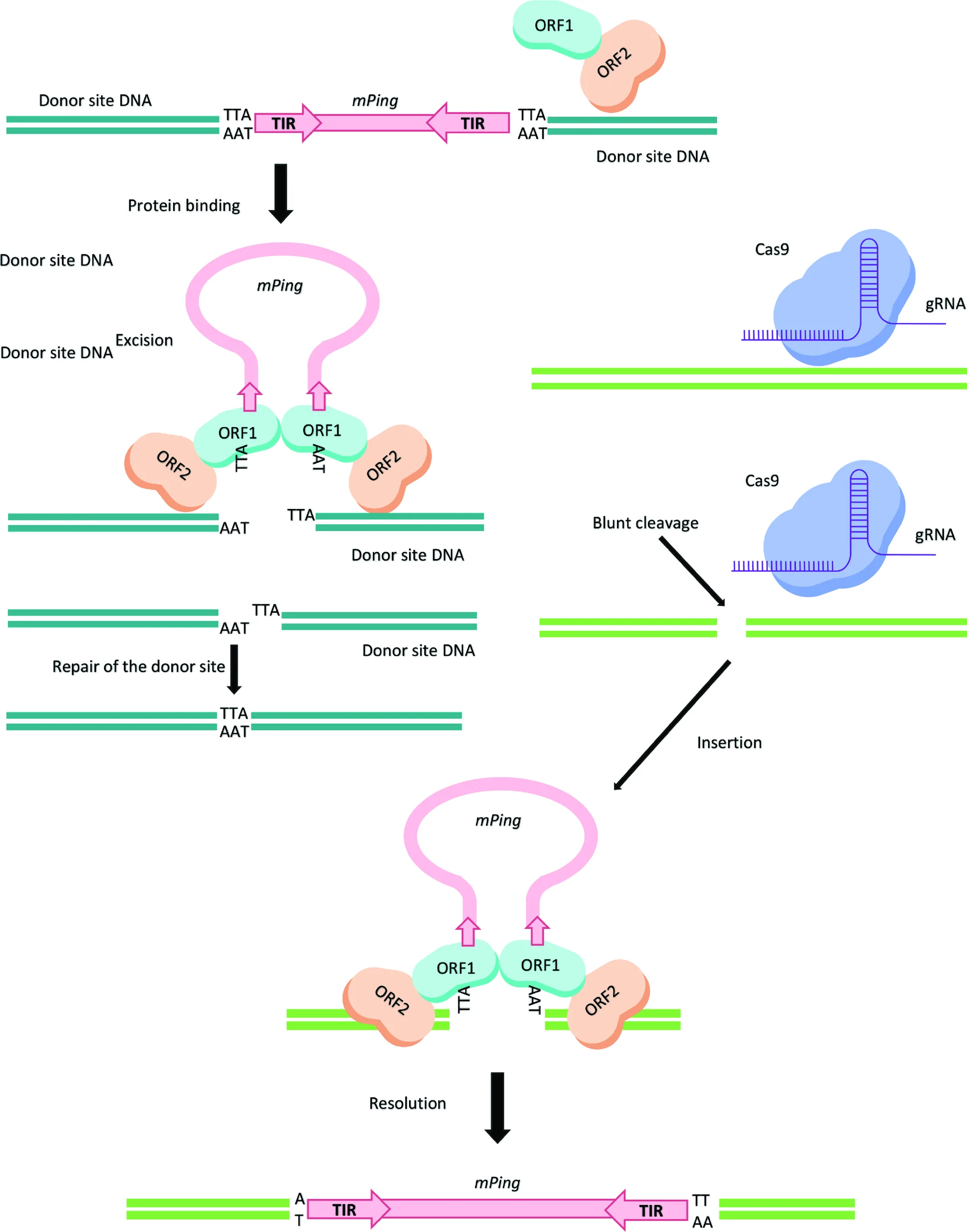
Model of excision and targeted insertion of mPing–Cas9. The open reading frame 1 (ORF1) and ORF2 proteins, encoded by the *Pong* transposon, associate with the *mPing* element to form a functional transposition complex. This complex is recruited to double-strand breaks generated by Cas9-mediated cleavage, where it catalyzes *mPing* integration. Mechanistically, ORF1 binds specifically to at least 15 bp of the *mPing* terminal inverted repeat (TIR) sequence, while ORF2 is responsible for the subsequent excision and insertion of the transposon. Excision occurs precisely at the flanking nucleotide sites (TTA or TAA) of the donor DNA.

The integration of large DNA fragments into specific genomic loci remains a central challenge in plant biotechnology. Transposons offer a natural tool for mobilizing DNA across genomes^[7]^. While transposons enable efficient, large-scale DNA integration, they typically insert randomly across the genome^[57]^. By contrast, CRISPR/Cas9 systems offer precise targeting but are limited to small edits or inefficient HDR for large insertions. Combining the cargo capacity of transposons with the targeting precision of Cas9 has emerged as a transformative strategy for programmable 'cut-and-paste' genome editing (Fig. 4).

Previous studies indicate that the hyperactive transposase encoded by the *Sleeping Beauty* transposon, when fused to dead Cas9 (dCas9, lacking cleavage activity), can be directed to specific genomic loci by guide RNAs to excise and insert genes of interest from donor plasmids. However, although this hybrid system targeted TTAA sequences when tested in animal cells, it failed to target specific sites^[67,68]^.

Systems like *mPing* (a miniature inverted-repeat TE) can deliver DNA fragments exceeding 10 kb in length into chromosomes but lack intrinsic targeting specificity, resulting in unpredictable insertion sites^[69]^. A system combining *mPing* with Cas9 (TATSI) achieved 26.7% targeted insertion efficiency in *Arabidopsis* for a 1-kb herbicide Bialaphos resistance gene (*Bar*). For large DNA cargo, TATSI reached up to 8.3% precise targeted insertion efficiency in *Arabidopsis* for an 8.6-kb insert and 9.8% in soybean (*Glycine max*) for a 1.5-kb herbicide-resistance gene^[13]^. Hybrid transposon–Cas9 systems represent a paradigm shift from random integration to programmable genome (re)writing. While challenges remain in efficiency and delivery, ongoing engineering efforts promise to unlock robust, scalable solutions for precise trait engineering in crops. As these tools mature, they will accelerate the development of climate-resilient, high-yielding varieties, underscoring their potential to revolutionize sustainable agriculture.

### R2Bm endonucleases from non–long terminal repeat (non-LTR) retrotransposons

Non–long terminal repeat (nLTR) retrotransposons, also known as retroposons, are ubiquitous components of eukaryotic genomes^[70]^. To date, 8,248 non-LTR retrotransposons have been identified^[63]^. Unlike their LTR-containing counterparts, they integrate without creating flanking repeats^[70]^. The R2 element is a well-studied model of site-specific non-LTR retrotransposons^[16,63]^. It is found exclusively within the *28S* ribosomal DNA (rDNA) genes of a wide range of animals and some protists, where it inserts into a conserved sequence with remarkable specificity^[63,66,71]^.

R2 elements are ancient, with homologous sequences identified in diverse metazoans, suggesting an origin predating the divergence of major animal phyla^[71]^. The entire R2 phylogenetic tree can be divided into 11 subclades, themselves defining four larger groups^[71]^. R2 elements were first discovered in the 1980s in the rDNA locus of the fruit fly (*Drosophila melanogaster*)^[72]^and later in the silkmoth *Bombyx mori*^[73]^.

The R2 element encodes a large, multi-domain enzyme (typically 1,000–1,200 aa) that integrates all activities necessary for retrotransposition^[74]^. The N-terminal domain is responsible for DNA binding and contains a CCHH-type zinc-finger motif critical for specific DNA binding to the *28S* rDNA target site^[16]^. This region is fused to an endonuclease domain related to the restriction enzyme–like GIV-YIG or PD-(D/E)xK superfamilies (varies by R2 lineage), catalyzing sequence-specific DNA cleavage^[16]^. The central reverse transcriptase (RT) domain is a canonical RT domain with the characteristic seven conserved motifs shared by other retroelement and telomerase RTs^[16]^. It catalyzes cDNA synthesis using the R2 mRNA as a template. The C-terminal domain is a poorly conserved region that may be involved in protein–protein interactions, nucleic-acid chaperone activity, or coordination with the other domains^[16]^. Its function is less defined but essential for activity.

While not as widely adopted as CRISPR/Cas systems, the R2 system offers unique advantages for engineered genome manipulation. Indeed, engineered R2 proteins with reprogrammed DNA-binding specificity can be used for site-specific gene insertions^[16,63]^, to integrate transgenes into a defined, safe genomic harbor (e.g., the highly repetitive *rDNA* locus). This approach is conceptually similar to CASTs but uses a distinct RNA/DNA template and integration biochemistry.

**Figure 5:**
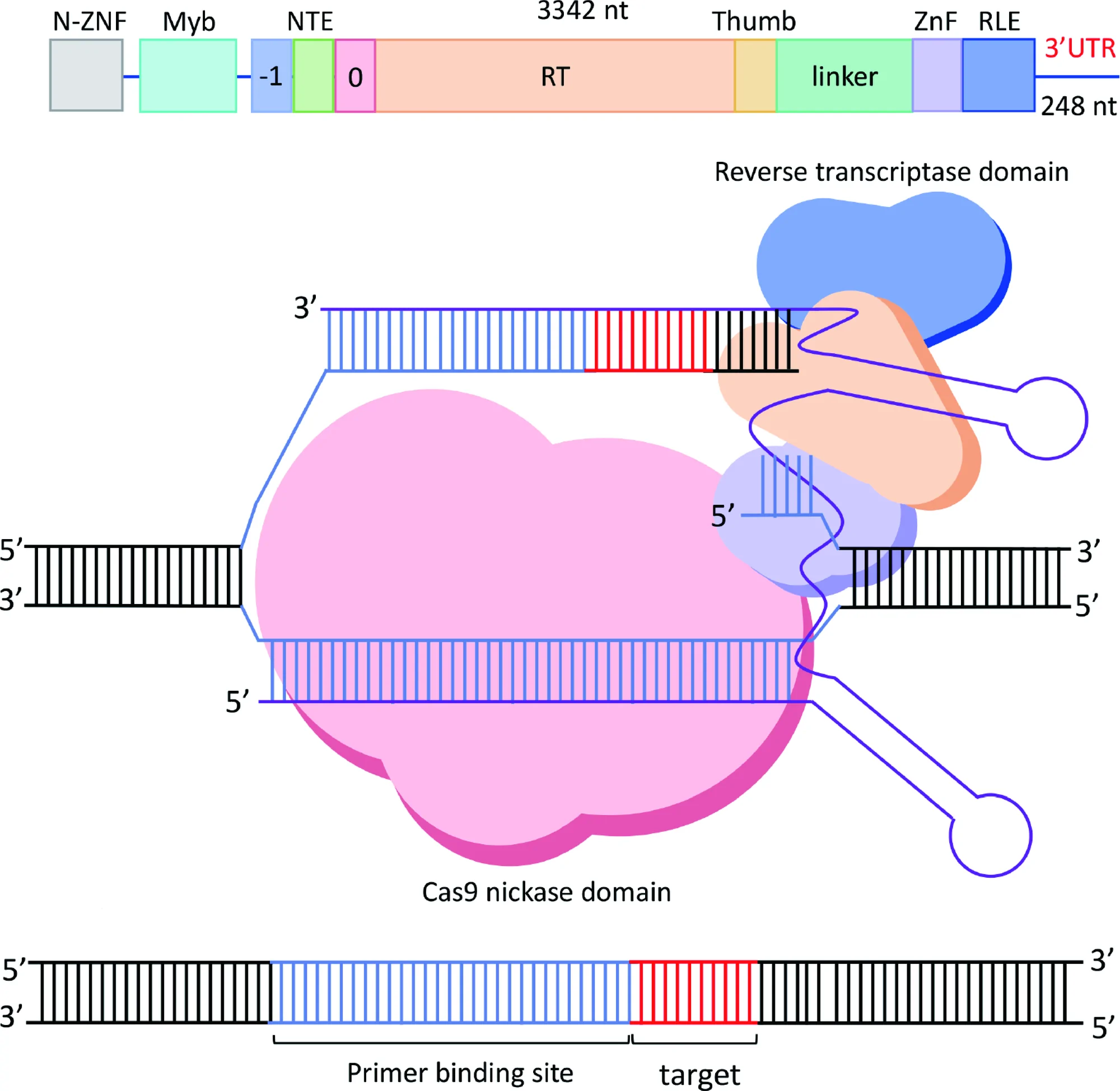
Domains of the R2Bm retrotransposon and diagram illustrating target-primed reverse transcription (TPRT). ZnF, zinc finger; NTE, N-terminal extension; R, reverse transcriptase; RLE, restriction-like endonuclease.

*In vitro* studies demonstrate that the R2Bm endonuclease from *B. mori*, when fused to dCas9, can be redirected by Cas9 guidance to perform target-primed reverse transcription (TPRT) at user-defined genomic loci independently of its native ribosomal target (Fig. 5). This engineered fusion protein enables the sequence-specific incorporation of small or large DNA fragments (up to ~300 bp) using a cargo RNA as template at these new target sites^[63]^.

Further optimization of the system, particularly using the engineered R2Tg variant from zebra finch (*Taeniopygia guttata*) has significantly enhanced integration efficiency. By chemically modifying the 5' end of the donor RNA and optimizing ribonucleoprotein (RNP) delivery, a compact RNP system was developed that achieves integration efficiencies exceeding 80% in several human cell lines^[66]^.

A refined version of this technology, termed STITCHR (site-specific target-primed insertion through targeted CRISPR homing of retrotransposons), utilizes the SpCas9^H840A^ nickase mutant fused to R2Tocc from white-throated sparrow (*Zonotrichia albicollis*). The STITCHR platform enables scarless and efficient genetic edits, ranging from 1-bp changes to insertions of large segments up to 12.7 kb. It supports applications such as precise gene replacement and can utilize *in* *vitro*-transcribed or synthetic RNA as templates, highlighting its versatility for programmable genome writing^[63]^. Notably, while STITCHR has shown efficient scarless DNA integration in human cells^[63]^, its feasibility and performance in plant systems remain to be explored (Table 3).

**Table 3:** Summary of the molecular and functional characteristics of programmable transposon systems.

System	CASTs (Type I–F)	CASTs (Type V–K)	TATSI (mPing-Cas9)	TPRT	STITCHR
Organism	Vibrionaceae^[14,62]^	Cyanobacteria^[15,62]^	Rice^[13]^	*Bombyx mori* ^[16]^	*Taeniopygia guttata* ^[63]^
Targeting strategy	RNA-guided^[14,62]^	RNA-guided^[15,62]^	RNA-guided^[13]^	RNA-guided^[16]^	RNA-guided^[63]^
Transposition mechanism	Cut-and-paste^[14,62]^	Cut-and-paste^[15,62]^	Cut-and-paste^[13]^	Cut-and-paste^[16]^	Cut-and-paste^[63]^
Targeting effector	Cas8–Cas5, Cas6, Cas7, and crRNA^[14,62]^	Cas12k, tracrRNA, and crRNA (sgRNA)^[15,62]^	Cas9, dCas9^[13]^	Cas9, dCas9^[16]^	Cas9, dCas9^[63]^
PAM	CC^[14,62]^	GT^[15,62]^	NGG^[13]^	NGG^[16]^	NGG
Transposase	TnsA and TnsB^[14,62]^	TnsB^[15,62]^	mPing^[13]^	R2 retrotransposon^[16]^	R2 retrotransposon^[63]^
Integration efficiency	100% in *E. coli* BL21(DE3); 1% in HEK293T cells^[57,62,64]^	95% in *E. coli* BL21(DE3); < 1% in HEK293T cells^[58,62,65]^	8.3% in *Arabidopsis* for 8.6-kb insert; 9.8% in soybean 9.8% for 1.5-kb insert^[13]^	< 300 bp *in vitro*^[16]^; 80% in human cells for 1.3-kb insert^[66]^	6%–12% in HEK293T cells for 1-bp to 12.7-kb insert^[63]^
Highlight	Highly specific targeting with complex effector composition	Compact yet sophisticated targeting effector composition	Insertion into specific target precisely	R2 can be retargeted with CRISPR/Cas9 and inserts small DNA fragments at the cleavage site *in vitro*	R2 can be retargeted with CRISPR/Cas9 and adds a single base to 12.7 kb DNA fragments at the cleavage site *in vivo*

### Merits of transposon-derived programmable nucleases

Genome-editing systems that use transposon-derived programmable nucleases offer distinct molecular and functional advantages, particularly in terms of protein compactness, guide RNA simplicity, PAM flexibility, cleavage pattern, and eukaryotic compatibility, which may address several limitations inherent to Cas9-based editing in plant and animal systems (Table 1). Transposons encode compact nucleases and boast enhanced deliverability. Cas9 orthologs (e.g., SpCas9) typically exceed 1,000 aa and thus place limits on the insert size that can be carried by the delivery vector. In contrast, TnpB, IscB, and Fz (~350–500 aa) may allow higher cargo capacity in vectors^[8−10,25,75]^. The small size of these systems permits packaging into viral vectors with restricted cargo capacity, broadening the range of *in vivo* and *in vitro* delivery strategies. In plants, compact nucleases are compatible with advanced nanoparticle carriers, viral vectors, and complex multigene assembly systems, thereby enhancing transformation efficiency and multiplex editing capabilities. Transposons also have relatively simple guide RNA systems. Unlike Cas9, which requires a dual-RNA complex (crRNA and tracrRNA) or a chimeric single guide RNA (sgRNA) with complex secondary structures, TnpB, IscB, and Fz utilize a single ωRNA for target recognition and nuclease recruitment. This simplified architecture reduces design complexity, enhances expression stability in eukaryotic cells, and minimizes off-target effects associated with sgRNA misfolding or suboptimal processing^[36,45,76]^. While transposons induce the formation of staggered DNA breaks, Cas9 generates blunt-ended DSBs, primarily repaired *via* error-prone non-homologous end joining (NHEJ). In contrast, TnpB and IscB produce 5′-staggered DSBs with short overhangs (typically 5–8 nt). This cleavage profile may favor alternative repair pathways such as MMEJ or HDR, potentially increasing the precision of knock-in edits and reducing unintended InDel formation. Fz represents the first known RNA-guided nuclease encoded by eukaryotic transposons. As such, it may exhibit improved folding, post-translational modifications critical for its function, and proper subcellular localization in eukaryotic environments. The compact and structurally distinct scaffolds of TnpB, IscB, and Fz provide new templates for engineering smaller, more specific, and multifunctional editors, such as compact base editors (BEs), prime editors (PEs), and transcriptional regulators. Their divergent evolutionary origins also offer opportunities to evade existing intellectual property constraints associated with CRISPR/Cas systems.

Although transposon-derived editors such as TnpB, IscB, and Fanzor have been shown to function in plants, their editing efficiencies remain substantially lower than those achieved by the established CRISPR/Cas9 systems. This performance gap likely reflects a combination of biological and technical constraints specific to plant systems.

First, suboptimal heterologous expression may limit nuclease activity^[77,78]^. As most transposon-derived nucleases originate from prokaryotic or non-plant eukaryotes, their coding sequences are rarely optimized for plant codon usage, potentially compromising translation efficiency and protein stability. Second, temperature sensitivity is another critical factor^[8]^. While many of these nucleases have been characterized under conditions optimal for bacterial or mammalian cells, plant transformation and regeneration are typically carried out at lower temperatures (22–28°C), which may reduce enzymatic activity. Third, plant chromatin architecture could impose additional restrictions^[79]^. Unlike Cas9, which has been extensively engineered to function across diverse chromatin states, transposon-derived editors may show reduced accessibility to compact or epigenetically silenced regions, thereby limiting editing efficiency. Fourth, guide RNA design and expression strategies remain underdeveloped. The ωRNA architecture, choice of promoters, and RNA stability in plant cells are not yet fully optimized, which may compromise target recognition and cleavage efficiency^[36]^. Furthermore, delivery and intracellular assembly of these systems may be less efficient in plant cells. Proper coordination between the nuclease protein and its cognate RNA component^[80]^, as well as efficient nuclear import, may differ markedly from animal systems.

Collectively, these observations indicate that the current editing inefficiencies are not due to inherent limitations of transposon-derived editors alone, but rather reflect their inadequate adaptation to the plant cellular environment. Systematic optimization of codon usage, thermal compatibility, RNA design, chromatin targeting, and delivery strategies will be essential to unlock the full potential of these emerging tools for plant genome engineering.

## High-efficiency plant regeneration: overcoming a key bottleneck for transposon-derived editing

Transposon-derived and large-cargo editors (e.g., CAST and R2 systems) expand the theoretical capacity for plant genome writing, but their practical deployment is constrained by delivery and regeneration bottlenecks. Thus, plant regeneration is not merely a downstream step but an integral part of the engineering pipeline. The large payloads of systems like CAST challenge size-limited viral vectors^[59,60]^, whereas Agrobacterium-mediated transformation offers higher cargo capacity, making it more suitable for delivering large constructs^[81]^. The Cut-Dip-Budding (CDB) system further bypasses tissue culture, enabling in planta regeneration from transformed cells—a key advantage for large-editor systems prone to low efficiency and transgene silencing. Coupling Agrobacterium delivery with CDB-based regeneration thus represents a promising strategy. Morphogenic regulators (e.g., BBM and WUS) can further enhance this process by promoting regeneration competence, increasing the likelihood that successfully transformed cells develop into whole plants. Collectively, the successful deployment of large-payload editing systems in plants will require co-optimization of editor design, delivery, and regeneration, with Agrobacterium and CDB playing central enabling roles.

### Phytohormonal calculus: auxin, cytokinin, and cell fate reprogramming

The foundation of *in vitro* plant regeneration was laid by the seminal discovery of Skoog and Miller that the balance of two phytohormones, namely auxin and cytokinin, dictates the developmental fate of cultured plant tissues^[82]^. A high auxin-to-cytokinin ratio typically induces root formation, while a high cytokinin-to-auxin ratio promotes shoot formation^[82]^.

Cytokinin-induced shoot regeneration involves changes in gene expression. The expression of cytokinin-responsive genes, in turn, is controlled by Type-B *ARABIDOPSIS* RESPONSE REGULATORs (ARRs), which bind to cytokinin response elements in the genes' promoters^[83−85]^. ARR10 and ARR12 directly activate the expression of *WUSCHEL* (*WUS*), which participates in the maintenance of the shoot apical meristem and in shoot regeneration^[84]^. ARR12 interacts with WUS RELATED HOMEOBOX 5 (WOX5) to suppress type-A *ARR* expression, thereby attenuating the negative feedback regulation of cytokinin signaling^[86]^. Another type-B ARR, ARR1, represses shoot regeneration through two parallel pathways: by competing with ARR12 for binding to the *CLAVATA 3* (*CLV3*) promoter to suppress *CLV3* expression, and by downregulating *INDOLE-3-ACETIC ACID INDUCIBLE 17* (*IAA17*) to indirectly inhibit *WUS* expression^[85]^.

Cytokinin signaling can be activated by wounding. The APETALA 2 (AP2)/ETHYLENE-RESPONSE FACTOR (ERF) transcription factors WOUND INDUCED DEDIFFERENTIATION 1 (WIND1), WIND2, WIND3, and WIND4 are the main modulators of the wounding response in *Arabidopsis* and are master regulators of cellular reprogramming; they enhance competence for subsequent fate changes, thus facilitating cell dedifferentiation before shoot regeneration^[87]^. WIND1 activates cytokinin production, enhances the B-Type ARR-mediated cytokinin responses, and activates CYCLIN D3 (CYCD3)^[88]^, thereby promoting cell dedifferentiation^[89]^. In addition, WIND1 also induces transcription of *ENHANCER OF SHOOT REGENERATION 1* (*ESR1*), involved in callus formation and *de novo* shoot organogenesis^[87]^. WIND1 induces the expression of *WOX13*, which encodes a direct regulator of *WIND2* and *WIND3* expression to induce cellular reprogramming^[90]^.

### Use of plant developmental regulators to promote regeneration

Many plant developmental regulators that enhance plant regeneration have been reported, e.g., LEAFY COTYLEDON 1 (LEC1)^[91]^, LEC2^[92]^, CLV3^[93]^, SHOOT MERISTEMLESS (STM)^[93]^, MONOPTEROS (MP)^[93]^, WUS^[94,95]^, BABYBOOM (BBM)^[95]^, GROWTH-REGULATING FACTOR 4 (GRF4), and GRF-INTERACTING FACTOR 1 (GIF1)^[96]^. The common characteristic of these proteins is that they promote somatic embryo production and/or shoot regeneration. Overexpression of *Bbm* or *Wus2* in maize (*Zea mays*) achieved high transformation frequencies in normally recalcitrant (poorly regenerating) maize inbred lines ^[97]^. Wang et al. reported a leaf-based transformation method using WUS2 and BBM for nine Poaceae species, including maize, sorghum, and foxtail millet, achieving efficient regeneration rates^[95]^. Overexpression of *Wus2* and *ISOPENTENYL TRANSFERASE* (*IPT*) or *BBM* in the leaves of *Nicotiana benthamiana* plants was sufficient to regenerate whole plants. When *Wus2* and *Bbm* were co-expressed with *Cas9* to edit the *Phytoene desaturase* (*PDS*) gene, the editing efficiency reached 15%^[98]^. Similarly, co-expression of *OsBBM1* and *OsWOX9A* in egg cells of rice resulted in 86%–91% fertilization-independent embryogenesis (parthenogenesis), representing a 4–15-fold increase over the rate obtained with *OsBBM1* expression alone^[99]^. A single *WUS* gene can also enhance plant regeneration efficiency. The wheat gene *TaWOX5* dramatically increases wheat immature embryo regeneration efficiency and partially overcomes genotype dependency^[100]^. The expression level of *WUS* may also affect the regeneration efficiency of apple (*Malus domestica*). Moderate expression of *MdWUS-1* significantly enhanced adventitious shoot regeneration from leaf explants, whereas its strong overexpression induced explant browning and necrosis, a phenotype potentially associated with oxidative stress^[101]^. The amount of cell proliferation induced by the combined expression of *PLETHORA 1* (*PLT1*) and *WOX5* varied with tissue and developmental stage in *Arabidopsis* and pepper^[102]^.

The co-expression of *GRF4* and *GIF1*, typically as a construct encoding a chimeric GRF4–GIF1 fusion protein, significantly enhances regeneration efficiency and speed in wheat, triticale, and rice. This system also expands the range of wheat genotypes amenable to transformation without introducing apparent developmental abnormalities. Notably, GRF4–GIF1 enables robust wheat regeneration in the absence of exogenous cytokinin, thereby allowing for the recovery of transgenic plants without the use of selectable markers. When integrated with CRISPR/Cas9 genome editing, the GRF4–GIF1 module facilitated the generation of successfully edited wheat plants^[96]^.

### The *Cut-Dip-Budding* (CDB) platform: a tissue-culture-free revolution

The Cut-Dip-Budding (CDB) system, first reported in 2022^[103]^, is a transformative *in planta* genetic transformation platform that circumvents the dependency on inefficient and genotype-limited tissue culture. Developed by Professor Jian-Kang Zhu's group^[103,104]^, CDB exploits the natural biology of *Agrobacterium rhizogenes*, which transfers Transfer-DNA (T-DNA) of Root-inducing (Ri) plasmid into wounded plant tissues, leading to the formation of abundant transgenic 'hairy roots' via the action of *rol* genes. However, the natural T-DNA from *A. rhizogenes* can cause Ri syndrome, and this trait may require segregation in subsequent generations, which poses a limitation for certain applications. The key innovation with CDB lies in harnessing the ability of these transgenic roots to spontaneously undergo developmental switching to produce adventitious shoots^[103]^, thereby enabling whole-plant recovery without the need for callus culture under sterile conditions. In addition, its application is limited to plant species that naturally produce adventitious shoots.

CDB demonstrates exceptional versatility across phylogenetically diverse species, including recalcitrant crops^[105]^, woody plants^[103]^, and ornamentals^[103]^ (Table 4). By unifying gene transfer, transgenic organogenesis, and plant regeneration into a single soil-based procedure, it offers a simple, rapid, and cost-effective alternative to conventional methods.

**Table 4:** Application of the CDB system in diverse plant species.

Plant type	Representative species	Transformation/editing efficiency	Key achievement	Ref.
Model and bioenergy dicots	Russian dandelion, *Coronilla varia*, *Platycodon grandifloras*, *Atractylodes macrocephala*, *Codonopsis pilosula*, Dihuang, Cannabis	High efficiency; demonstrated CRISPR/Cas9 editing	Established CDB as a rapid, efficient platform for root-based metabolic engineering	[103,105, 112,113]
Tuber crops	Sweetpotato (*Ipomoea batatas*)	High; CRISPR-mediated editing efficiency up to 100% in some cultivars	Overcame severe genotype dependence, enabling editing of multiple farmer-preferred varieties	[103]
Woody perennials	*Ailanthus altissima*, *Aralia elata*, *Clerodendrum chinense*	Moderate to high transformation rates	Provided a viable transformation method for tree species where tissue culture is extremely difficult or non-existent	[103]
Succulent dicots	Kalanchoe (*Kalanchoe blossfeldiana*)	Exceptionally high, up to 74% transformation efficiency and 70% editing efficiency	Achieved the highest reported efficiency for any succulent, enabling functional genomics in ornamentals	[104]
Succulent monocots	Snake plant (*Sansevieria trifasciata*)	Moderate, ranging from 3.9% to 7.8%	Reported the first-ever efficient genetic transformation system for this globally important ornamental monocot	[104]

Seven common strains of *R. rhizogenes* (K599, MUS440, A4, AQ, AR1193, C58 C1, and ATCC) have been used in crop regeneration in combination with the CDB system. Among these, strain K599 has been extensively studied. When inoculated on soybean hypocotyls, nearly 100% of infected seedlings produced transgenic positive roots by 16 d post-inoculation across seven tested genotypes, achieving a transformation efficiency three times higher than that of traditional *A. rhizogenes*-mediated transformation^[106]^. In *Phaseolus vulgaris* seedlings, infection with K599 resulted in a transformation rate of up to 35%, and Cas9-induced mutations were observed in 70% of the transgenic events^[107]^.

Strain K599 has also been used in various woody plant species. It successfully induced hairy roots in diverse citrus cultivars, including grapefruit (Rio Red, *Citrus* × *paradisi* Macf), sweet orange (Valencia, *Citrus* × *sinensis*), rough lemon (*Citrus* × *jambhiri* Lush), carrizo (*Citrus* × *poncirus*), and citron (*Citrus medica*), with efficiencies ranging from 28% to 75%. The induced hairy roots could be regenerated into transgenic plants by supplementing plant hormones^[108]^.

In apple (*Malus pumila*) and kiwifruit (*Actinidia chinensis*), K599-mediated transformation achieved 78.8% efficiency for transgenic hairy root generation, with a root-to-shoot regeneration efficiency of 3.3%. Under this protocol, transgenic plants were obtained within approximately 14 weeks^[109]^. In tea plants, another strain, ATCC, achieved a transformation rate of up to 14.2%^[110]^.

In cannabis (*Cannabis sativa*), strain A4 produced the highest transgenic hairy root efficiency, reaching up to 75%, while the ARqual strain yielded the lowest efficiency at 8.33%, and K599 failed to induce transformed roots. Importantly, transformation efficiency was not affected by the cannabis chemotype (hemp-type vs. drug-type)^[105]^. Strain MUS440 was also reported to generate transgenic and gene-edited hairy roots in radish (*Raphanus sativus* L.)^[111]^.

This system is positioned as a powerful tool for functional genomics, CRISPR/Cas editing, and the biotechnological improvement of a wide range of previously difficult-to-transform plants.

## Conclusion and future perspectives

Realizing the full potential of transposon-derived genome editors in plants requires focused advances on several interdependent fronts. A primary challenge is enhancing editing efficiency and specificity. Meeting this goal necessitates continued engineering of TnpB, IscB, and Fz nucleases and ωRNA through structure-guided design and directed evolution, alongside comprehensive off-target profiling to develop high-fidelity variants. For large-fragment integration systems like CAST, optimizing the stoichiometry and functional coordination of their multi-protein complexes in plant cells is paramount. Concurrently, functional diversification, through metagenomic discovery of as-yet-undescribed editors and engineering of compact base or prime editors, will significantly broaden the application scope.

Systematic tool development and benchmarking are urgently needed, including standardized vectors, validated ωRNA design tools, and public databases, coupled with rigorous comparative analysis of different systems. Critically, the utility of these editors, especially for large DNA delivery, is limited by the difficulty of plant regeneration. Future strategies must co-design editing and regeneration systems, employing vectors that couple editors with inducible morphogenic regulators (e.g., BBM, WUS), leveraging *in planta* platforms like CDB, or engineering elite recipient genotypes. Furthermore, exploiting systems with unique capabilities, such as eukaryotic Fz for organelle editing or R2-based STITCHR for long cDNA integration, will open new application spaces.

In conclusion, the future of plant genome writing lies in a convergent, modular toolkit. Transposon-derived systems are pivotal, compact modules for editing and targeted DNA integration. Their success depends on synergistic integration with advances in protein design, delivery technology, and synthetic biology, ultimately enabling the precise rewriting of plant genomes for transformative crop improvement.

## Data Availability

Data sharing is not applicable to this article as no datasets were generated or analyzed during the current study.
